# Li_4_Ti_5_O_12_/graphene nanoribbons composite as anodes for lithium ion batteries

**DOI:** 10.1186/s40064-015-1438-0

**Published:** 2015-10-26

**Authors:** P. A. Medina, H. Zheng, B. D. Fahlman, P. Annamalai, A. Swartbooi, L. le Roux, M. K. Mathe

**Affiliations:** Department of Chemistry and Science of Advanced Materials Program, Central Michigan University, Mount pleasant, Michigan, 48858 USA; Material Science and Manufacturing, Council for Scientific and Industrial Research (CSIR), PO BOX 395, Pretoria, 0001 South Africa

**Keywords:** LIBs, Li_4_Ti_5_O_12_, Graphene nanoribbons, Anode, Capacity

## Abstract

In this paper, we report the synthesis of a Li_4_Ti_5_O_12_/Graphene Nanoribbons (LTO/GNRs) composite using a solid-coating method. Electron microscope images of the LTO/GNRs composite have shown that LTO particles were wrapped around graphene nanoribbons. The introduction of GNRs was observed to have significantly improved the rate performance of LTO/GNTs. The specific capacities determined of the obtained composite at rates of 0.2, 0.5, 1, 2, and 5 C are 206.5, 200.9, 188, 178.1 and 142.3 mAh·g^−1^, respectively. This is significantly higher than those of pure LTO (169.1, 160, 150, 106 and 71.1 mAh·g^−1^, respectively) especially at high rate (2 and 5 C). The LTO/GNRs also shows better cycling stability at high rates. Enhanced conductivity of LTO/GNRs contributed from the GNR frameworks accelerated the kinetics of lithium intercalation/deintercalation in LIBs that also leads to excellent rate capacity of LTO/GNRs. This is attributed to its lower charge-transfer resistance (Rct = 23.38 Ω) compared with LTO (108.05 Ω), and higher exchange current density (j = 1.1 × 10^−3^ mA cm^−2^)—about 20 times than those of the LTO (j = 2.38 × 10^−4^ mA cm^−2^).

## Background

As a most effective and practical technology, lithium ion batteries (LIBs) continue to provide a promising solution to increasing energy demands of portable devices such as mobile phones and laptops. LIBs are also explored as promising for electric vehicles (EVs), hybrid electric vehicles (HEVs) and energy storage systems in terms of their high power density, long cycle life and high safety(Armand and Tarascon 2008; Scrosati and Garche 2010).

Lithium titanate (Li_4_Ti_5_O_12_, LTO) is regarded as a favorable anode material, since the traditional carbon/graphite materials have shown some critical issues including poor cyclic life; and high reactivity with the electrolyte solution that easily contributes to the thermal runaway of batteries under certain reported conditions (Yao et al. 2005). Li_4_Ti_5_O_12_ anode material is a zero-strain insertion material whose lattice dimension does not change during charge/discharge processes, and is thus ideal for long-life rechargeable batteries (Ohzuku et al. 1995). LTO with a theoretical capacity of 175 mAh·g^−1^ has excellent Li^+^ insertion and extraction reversibility in the voltage range of 1.0–2.5 V. Additionally, LTO has a very flat voltage plateau close to 1.55 V (vs. Li/Li^+^), which sufficiently avoids the formation of metallic lithium, thus resulting in improvement of the safety of lithium-ion batteries (Zhang and Li 2013). However, one practical problem associated with unmodified Li_4_Ti_5_O_12_ is its poor rate performance, resulting from its inherent low electronic conductivity and moderate Li^+^ diffusion coefficient (Kavan et al. 2003; Wagemaker et al. 2008; Ouyang et al. 2007). Numerous strategies amongst others have been attempted to improve Li_4_Ti_5_O_12_ such as tailoring the particle size of the materials to the nanometer-scale, doping with other elements, and coating with different carbon materials (Zhang and Li 2013; Jung et al. 2011; Hao et al. 2005; Kim et al. 2010; Sorensen et al. 2006; Yi et al. 2010).

Graphene nanoribbons (GNRs) are strips of graphene with outstanding electronic properties. GNRs have been used in a wide range of device materials (Han et al. 2007; Jian et al. 2012; Kosynkin et al. 2009; Jiao et al. 2009). Recent theoretical and experimental studies have shown that GNRs can enhance lithium storage capacity through edge effect (Uthaisar et al. 2009; Bhardwaj et al. 2010). On the basis of the unzipping mechanism (Uthaisar et al. 2009; Bhardwaj et al. 2010), a large number of edge sites are created during the formation of GNRs, which might produce more electrochemically active sites for charge transfer during charge/discharge when compared to graphene and carbon nanotubes. Moreover, GNRs, having large aspect ratios and high surface area, might provide an excellent conductive matrix with good mechanical flexibility for anode or cathode materials to accommodate the volume changes during change/discharge cycles. GNRs composited with SnO_2_ (Dong et al. 2015), Fe_3_O_4_ (Lin et al. 2014) and MnO_2_ (Li et al. 2013) have been reported as anodes, with V_2_O_5_ (Yang et al. 2014) as cathodes in LIBs. Those results have demonstrated that the formation of more edge sites in GNRs does not lead to a loss in the conductivity of the composites. However, to date, the use of GNRs to stimulate enhanced LTO electrochemical performance for LIBs has not been reported. In this work, the LTO anode performance for LIBs using GNRs as additive is investigated and reported as a first.

## Experimental

### Synthesis of materials

A modified Hummer’s method was used to synthesize graphene oxide nanoribbons (GONRs) (Hummers and Offeman 1958). Firstly, 600 mg of MWCNTs (50–80 nm, Cheap Tubes Inc, Cambridgeport, VT, USA) were suspended in 144 mL of sulfuric acid (H_2_SO_4_) (98 %, Aldrich) and stirred for 1 h. Then, 16 mL of concentrated H_3_PO_4_ was added with further stirring for 15 min. Following the stirring, 300 mg of KMnO_4_ (99.9 %, Aldrich) was slowly added to the mixture with the reaction taking place for 2 h at 65 °C. The resultant product waspoured over 400 mL of ice, with 20 mL of H_2_O_2_ (30 %, ACE, South Africa). The product was cooled to room temperature then filtered using a 200 nm pore size PTFE membrane. The product was washed sequentially with 30 % HCl (24 mL each), ethanol (ACE, South Africa) and ether (ACE, South Africa). Lastly, the black solid product was dried at 90 °C overnight under vacuum.

The GONRs formed from the above process was then reduced thermally. Using a tube furnace, a temperature of 250 °C was reached before a 1:1 Ar:H_2_ gaseous mixture was introduced and maintained for 1 hour.

Anatase TiO_2_ (220 nm in average particle diameter and 99.5 % in purity, Hangzhou Wanjing New Material Co., Ltd) and Li_2_CO_3_ (99.9 % in purity, Shanghai China Lithium Industrial Co., Ltd.) were used as raw materials. Li_4_Ti_5_O_12_ was prepared by a solid-state reaction method. The stoichiometric amounts of Li_2_CO_3_ and anatase TiO_2_ with molar ratio of Li:Ti = 0.82:1 were mixed with ethanol as dispersant by planetary ball-milling. The ball-milled mixture was heated in a furnace at 800 °C in air for 18 h, followed by mechanical crushing to obtain the final Li_4_Ti_5_O_12_.

LTO/GNRs (5 % wt GNRs) composites was formed by mechanical mixing method.

### Characterization

The morphological and structural properties of the GNRs were determined using X-ray diffraction (XRD, SCINTAG-XDS 2000), transmission electron microscopy (TEM, Hitachi H-7000 200 kV), and scanning electron microscopy coupled with energy dispersive spectroscopy (SEM/EDS) (JSM-7500F SEM/EDS). Battery performance was conducted on a Maccor testing system. Electrochemical performance was tested with a Biologic potentiostat.

### Coin cells assembly and electrochemical measurements

LTO or LTO/GNRs was dispersed homogeneously in a slurry with 10 % poly (vinylidene fluoride) binder with 10 % PRINTEX XE 2-B in N-methylpyrrolidione (NMP) solvent. The slurry was subsequently cast onto a Cu foil current collector and then dried overnight under vacuum at 110 °C. In the coin cell, metallic lithium foil was used as the counter and reference electrodes; the electrolyte used was a 1 mol/L LiPF_6_ in a 1:1 solvent mixture of ethylene carbonate and diethyl carbonate (EC/DEC). Electrochemical measurements were carried out between 1.0 and 2.5 V vs Li^+^/Li^0^ with CR2032 coin cells. The capacity of the cell was calculated on the basis of the total mass of the active material (LTO).

## Results and discussion

### Structure and morphology

**Fig. 1:**
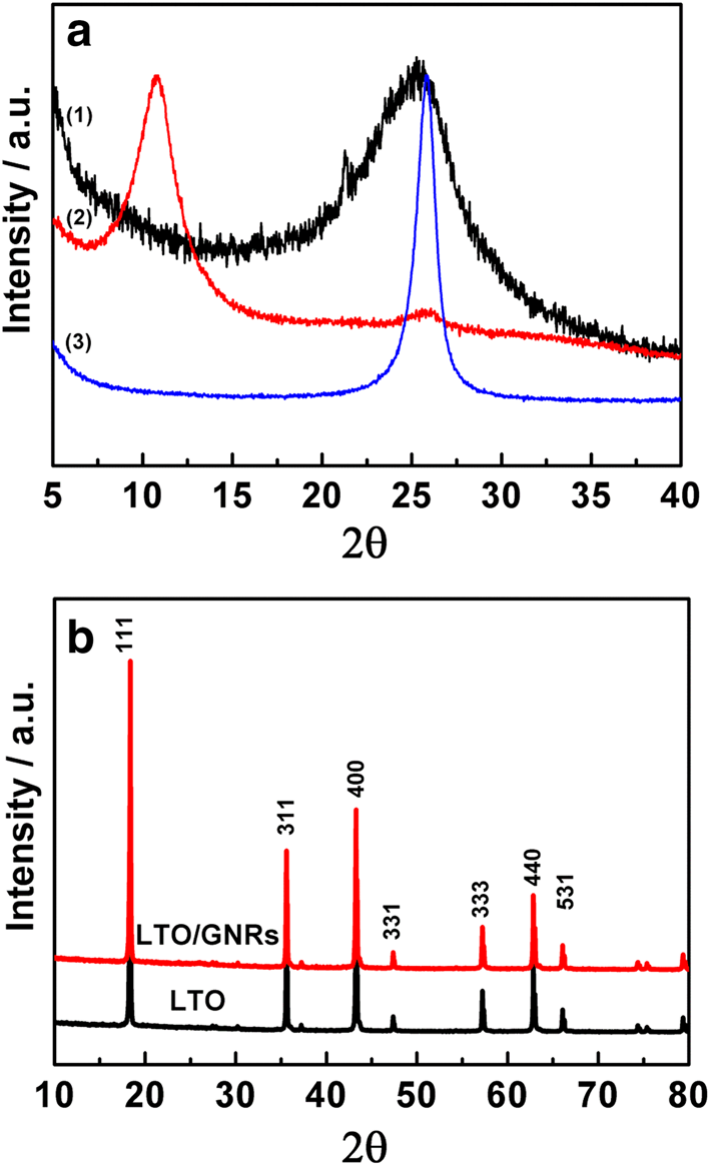
XRD pattern. **a** For GNRs (1), GNORs (2) and MWCNTs (3) and **b** for LTO and LTO/GNRs

The XRD micrographs of graphene oxide nanoribbons (GONRs), thermally-reduced graphene nanoribbons (GNRs), LTO, and LTO/GNRs materials were generated and are presented in Fig. 1. The GONRs (“2”) exhibited a highly crystalline structure, with a pronounced peak at 2θ = 10.8° including a small peak at 2θ = 25.6°, indicating that traces of the starting material of carbon nanotubes (“3”) were still present in the sample. Figure 1a also displays XRD of the GNRs materials reduced from GONRs (“1”) without the peak at 2θ = 10.8°. The peak at 2θ = 25.2° suggests that GONRs were completely reduced to a graphite-like structure. Peaks for GNRs were absent in LTO/GNRs composites, and have shown the same typical peaks with spinel LTO as can be seen in Fig. 1b. This suggests that the small amount of GNRs added were completely covered by LTO particles, and were thus overlapped by the strong XRD peaks of spinel LTO.

**Fig. 2:**
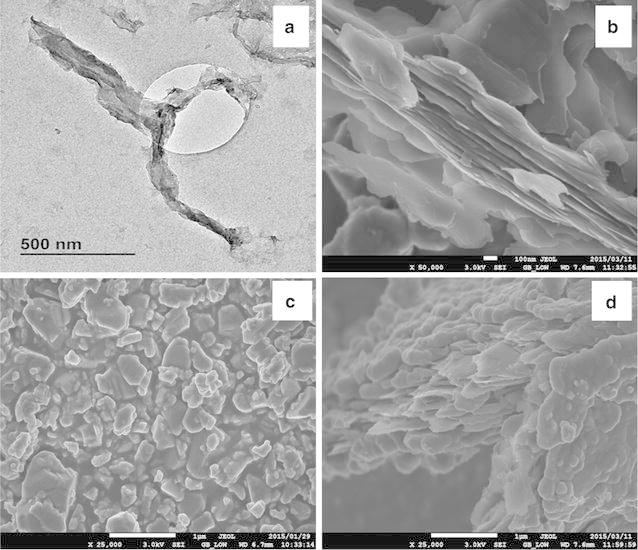
TEM images of GNRs (**a**), SEM images of GNRs (**b**), LTO (**c**) and LTO/GNRs (**d**) materials

Figure 2 shows both TEM and SEM structures of GONRs, LTO and LTO/GNRs. As observed from the TEM image in Fig. 2a, MWNTs were completely unzipped, resulting in wavy-structure. GNRs strips shown in Fig. 2a had an average width of 75–150 nm. The typical unzipped nanoribbons with multi-layered structures are as shown by SEM images (Fig. 2b). In contrast, the LTO particlesof a size range from 100–800 nm are presented (Fig. 2c). It is worth noting from LTO particles in Fig. 2d that these were wrapped around graphene nanoribbons.

### Electrochemical performances

**Fig. 3:**
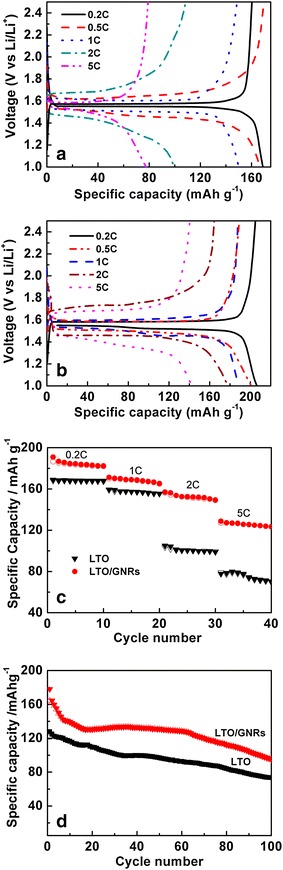
Initial discharge/charge voltage vs. capacities at different rates on LTO (**a**), LTO/GNRs (**b**) electrodes, the rate performance (**c**) and cycling performance (**d**) of LTO and LTO/GNRs

The electrochemical behaviour of the LTO/GNRs sample was tested at various charge/discharge rates from 0.2 to 5 C in the potential range of 1–2.5 V vs. Li/Li^+^. The anodic performance of LTO and LTO/GNRs are indicated in Fig. 3 with a plot of initial capacity at different charge/discharge rates. When comparing Fig. 3a, b, both electrodes display flat operation potential plateaus at low rates; for example, 0.2, 0.5 and 1 C. However, when increasing the rate, the potential plateaus of the LTO became shorter and gradually bent. This could result from an increase of polarization on pure LTO at higher rate, while that of the LTO/GNRs electrode still remains flat at 2 and 5 C. At low rates of 0.2, 0.5 and 1 C, the specific capacities of LTO/GNRs were 206.5, 200.9 and 188 mAh·g^−1^, respectively. This is higher than that of the LTO (169.1, 160 and 150 mAh·g^−1^, respectively). The initial discharge capacities of LTO/GNRs at 0.2–1 C were higher than the theoretical capacity of 175 mAh·g^−1^, which indicates the existence of additional lithium storage sites at the LTO/GNRs electrode (Sun et al. 2014). At 0.5 C, the capacity on LTO/graphene was reported as 177 mAh·g^−1^ (Ri et al. 2013***)***, which is slightly lower than that achieved from LTO/GNRs at 200.9 mAh·g^−1^. Moreover, the relative difference in specific capacity was particularly larger at higher rates when LTO was added to GNRs. In particular, at high charge/discharge rates of 2 and 5 C, the specific capacities of LTO/GNRs were 178.1 and 142.3 mAh·g^−1^, respectively—much higher than that of the LTO alone (106 and 71.1 mAh·g^−1^, respectively). This also indicates that the LTO/GNRs electrode has lower polarization due to the improved electrical conductivity produced by the GNRs. Figure 3c depicts different charge–discharge rates used to study the electrochemical stability of LTO and LTO/GNRs. Ten cycles of the charge/discharge process are included for each rate. Figure 3c shows that LTO/GNRs has more degradation per cycle of capacity from 0.2–1 C (1.3 mAh·g^−1^/per cycle) than that of LTO (0.7 mAh·g^−1^/per cycle). This capacity degradation is possibly because LTO/GNRs had suffered from volume change of GNRs additive during discharge/charge process. However, the discharge capacity of LTO/GNRs has high retention at high rates from 2 to 5 C. The discharge capacity of LTO/GNRs still remains at 123 mAh·g^−1^ after 5 C, whereas that of LTO is only 70.3 mAh·g^−1^.

The cycling performance of LTO and LTO/GNRs were tested at a 2 C rate for 100 cycles. It is seen from Fig. 3d that the initial discharge capacities of the LTO and LTO/GNRs were 121.4 and 178.2 mAh·g^−1^, respectively, and the retained discharge capacities were 73.4 and 94.7 mAh·g^−1^ after 100 cycles. The excellent cyclic stability and high-rate capacity of LTO/GNRs could be related to increased conductivity of LTO/GNRs.

**Fig. 4:**
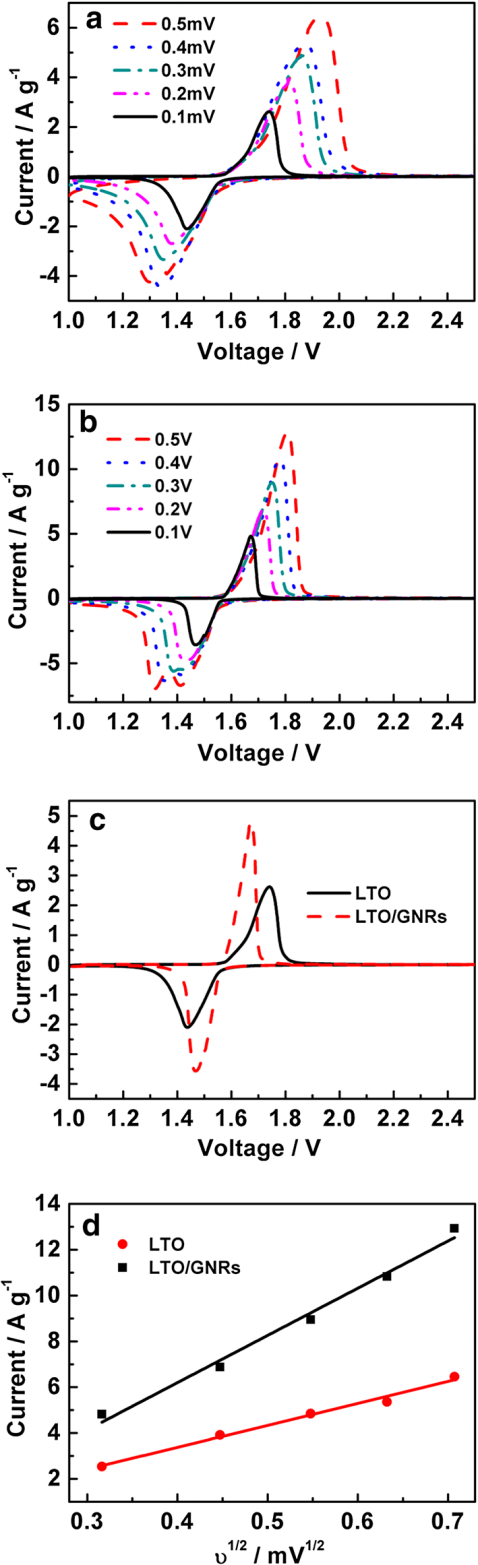
CV of LTO (**a**) and LTO/GNRs (**b**) at different scan rate; comparison of both electrodes at scan rate of 01 mV s^−1^ (**c**); The linear relations of peak current and the square root of the scan rate (υ^1/2^) (**d**)

To better understand the high rate charge/discharge property of the LTO/GNRs, cyclic voltammograms were generated at various scan rates for LTO/GNRs and LTO and are presented in Fig. 4. At a scan rate of 0.1 mVs^−1^ (Fig. 4c), both electrodes have redox couple peaks at around (1.46, 1.7 V), which corresponds to the lithium ion intercalation/de-intercalation of LTO. It should be noted that the peak for LTO/GNRs was sharper, and the gap between redox peaks was smaller than that of pristine LTO, indicating that the former had a lower overall resistance and higher kinetics for the redox reaction (Li et al. 2013; Goriparti et al. 2014).

The anode peak of LTO/GNRs is negatively shifted by 70 mV (from Fig. 4c) with almost double the current density compared with LTO. This further suggests that LTO/GNRs has a better electrochemical performance than that of LTO. It was observed that with increasing the scan rate, the intercalation and de-intercalation peaks shift to lower and higher potentials, respectively. However, smaller potentials shifts were noticed for LTO/GNRs than that of LTO between intercalation and de-intercalation peaks, thus showing improved kinetics by incorporating cathodically-induced graphene nanoribbons. As displayed in Fig. 4d, the peak current (j) exhibits a linear relation with the square root of the scan rate (ʋ^1/2^), which indicates a diffusion-controlled process rather a surface-controlled one (Li et al. 2013).

**Fig. 5:**
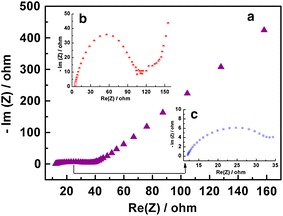
Electrochemical impedance spectroscopy of the LTO/GNRs (**a**, **c**) and the LTO (**b**) electrode

The results of the electrochemical impedance spectroscopy (EIS) are presented in Fig. 5. The LTO/GNRs electrode shows a much lower charge-transfer resistance than that of the pristine LTO electrode [23.38 (c) vs. 108.05 Ω (b)] on the basis of the equivalent circuit given by the inset of Fig. 5. Furthermore, the lower charge-transfer resistance of the LTO/GNRs cell led to higher exchange current densities based on the formula (j = RT/nFRct) (see Table 1). A higher exchange current density (j = 1.1 × 10^−3^ mA cm^2^) was obtained on LTO/GNRs electrode, which is about 20 times that of LTO (j = 2.38 × 10^−4^ mA cm^2^). The faster charge-transfer kinetics of the LTO/GNRs electrode could be attributed to GNRs, which enhanced the conductivity of the electrode.

**Table 1:** Electrochemical properties of the LTO/GNRs and the LTO electrode

Samples	R_ct_/Ω	J/mA cm^−2^
LTO	108.05	2.38 × 10^−4^
LTO/GNRs	23.38	1.1 × 10^−3^

## Conclusions

In summary, LTO/GNRs composites were shown to provide improved discharge capacities of 206.5 and 142.3 mAh·g^−1^ at 0.2 and 5 C, respectively, which is higher than those of pristine LTO (169.1 and 71.1 mAh·g^−1^). Therefore, GNRs additives were proved to have improved the LTO rate performance, especially at high rates. The enhanced conductivity LTO/GNRs, which are contributed from the GNR Framework and high conductivity, results in accelerated kinetics for lithium intercalation/de-intercalation within LIBs that also leads to excellent rate capacity of LTO/GNRs.
